# Study of SV40 large T antigen nucleotide specificity for DNA unwinding

**DOI:** 10.1186/s12985-017-0733-5

**Published:** 2017-04-14

**Authors:** Damian Wang, Ana Lucia Álvarez-Cabrera, Xiaojiang S. Chen

**Affiliations:** 1 Genetic, Molecular, and Cellular Biology Program, Keck School of Medicine, University of Southern California, Los Angeles, 90033 CA USA; 2 Molecular and Computational Biology Program, Departments of Biological Sciences and Chemistry, University of Southern California, Los Angeles, 90089 CA USA; 3 Center of Excellence in NanoBiophysics, University of Southern California, Los Angeles, 90089 CA USA; 4 Norris Comprehensive Cancer Center, University of Southern California, Los Angeles, 90089 CA USA

**Keywords:** SV40 virus, SV40 large t antigen, Replicative helicase, DNA replication, Nucleotide binding and hydrolysis, Nucleotide specificity for unwinding

## Abstract

**Background:**

Simian Virus 40 (SV40) Large Tumor Antigen (LT) is an essential enzyme that plays a vital role in viral DNA replication in mammalian cells. As a replicative helicase and initiator, LT assembles as a double-hexamer at the SV40 origin to initiate genomic replication. In this process, LT converts the chemical energy from ATP binding and hydrolysis into the mechanical work required for unwinding replication forks. It has been demonstrated that even though LT primarily utilizes ATP to unwind DNA, other NTPs can also support low DNA helicase activity. Despite previous studies on specific LT residues involved in ATP hydrolysis, no systematic study has been done to elucidate the residues participating in the selective usage of different nucleotides by LT. In this study, we performed a systematic mutational analysis around the nucleotide pocket and identified residues regulating the specificity for ATP, TTP and UTP in LT DNA unwinding.

**Methods:**

We performed site-directed mutagenesis to generate 16 LT nucleotide pocket mutants and characterized each mutant’s ability to unwind double-stranded DNA, oligomerize, and bind different nucleotides using helicase assays, size-exclusion chromatography, and isothermal titration calorimetry, respectively.

**Results:**

We identified four residues in the nucleotide pocket of LT, cS430, tK419, cW393 and cL557 that selectively displayed more profound impact on using certain nucleotides for LT DNA helicase activity.

**Conclusion:**

Little is known regarding the mechanisms of nucleotide specificity in SV40 LT DNA unwinding despite the abundance of information available for understanding LT nucleotide hydrolysis. The systematic residue analysis performed in this report provides significant insight into the selective usage of different nucleotides in LT helicase activity, increasing our understanding of how LT may structurally prefer different energy sources for its various targeted cellular activities.

## Background

Helicases are molecular machines that use energy obtained from nucleotide triphosphate (NTP) binding and hydrolysis to unwind duplex DNA—a fundamental process in genomic replication [1]. Simian Virus 40 (SV40) encoded Large Tumor Antigen (LT) is a superfamily III helicase, belonging to the AAA+ (*A*TPase *a*ssociated with various cellular *a*ctivities) family of proteins and contains 708 amino acids that fold into multiple domains. LT is a multifunctional protein that not only behaves as a potent oncoprotein (reviewed in [2–7] and references therein), but functions as an efficient DNA helicase to melt double-stranded origin DNA and unwind fork DNA for viral replication utilizing ATP as the primary energy source [6, 8–12]. Its major functional domains for replication, contained in residues 131–627 [6, 12–17], includes an origin DNA binding domain (OBD; residues 131–259), a zinc coordinating domain, and its modified Walker A (P-loop), Walker B motifs and arginine fingers responsible for its nature as an ATPase [17–19]. SV40 has been extensively used as a paradigm for studying eukaryotic DNA replication due to its well-defined origin of replication and dependence on LT alone for assembling at the origin to initiate melting for viral genomic replication [4, 6, 12, 20].

**Fig. 1:**
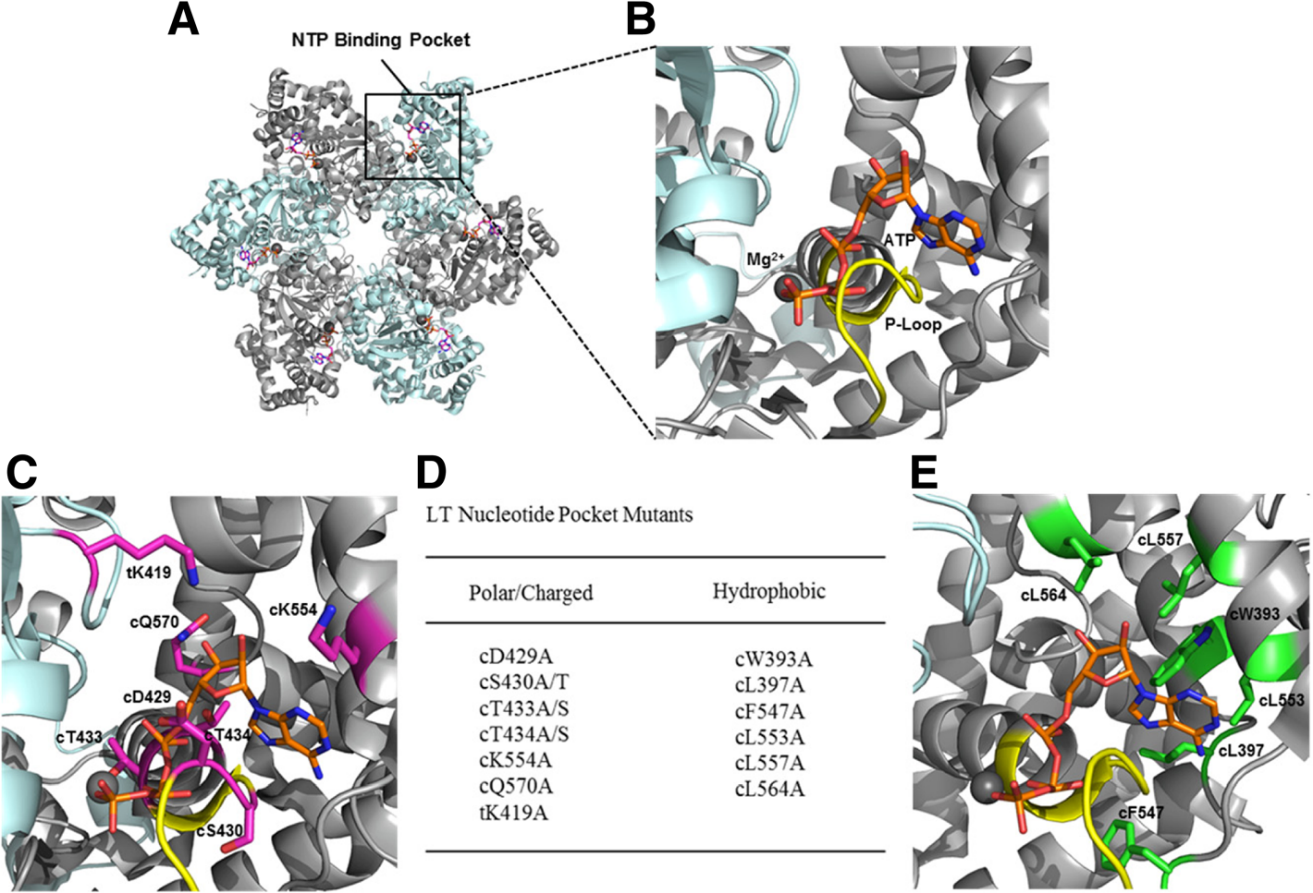
Structure of the SV40 LT nucleotide binding pocket and the various pocket mutants. **a** Crystal structure of the LT hexameric helicase, represented by residues 251–627 in the form of a hexamer bound to six ATP (PDBid: 1SVM). The nucleotide pocket is located at the interface between adjacent monomers as indicated with an outlined *box*. **b** A close-up of the nucleotide pocket bound to ATP and Mg^++^. The P-loop is colored in *yellow*. **c**, **d**, **e** Locations of the polar/charged residues **c** and hydrophobic residues **e** chosen for the mutational analysis of the binding pocket are indicated in *magenta* and *green* and their mutants are listed in **d**

Upon binding to ATP in the presence of Mg^++^, LT assembles into ring-shaped hexamers [15, 21, 22], establishing the functional unit in coupling energy produced by ATP binding and hydrolysis to the mechanical work needed for double-stranded DNA (dsDNA) unwinding. The six monomeric units that form the higher order hexamer also generate the six active NTP binding pockets located at the interface between adjacent monomers (Fig. 1a, b), with the cis- (c) monomer being the subunit with NTP bound to its P-loop, and the trans- (t) monomer being the subunit supplying the arginine finger residues for hydrolysis. As a result, the amino acid residues within the interface of both the cis- (c) and trans- (t) monomeric units play important roles in NTP binding and ensuing hydrolysis [15, 23].

Although LT helicase activity has primarily been coupled to the hydrolysis of ATP [9], earlier studies showed that immunopurified full-length LT could also coordinate the hydrolysis of UTP, CTP, and GTP, as well as their corresponding deoxynucleotide triphosphates (dNTPs) to support lower levels of DNA unwinding activity [24]. Additionally, it has been demonstrated that dCTP, UTP, and TTP could also, to a lesser extent, stimulate the binding LT to the SV40 core origin of replication sequence [25]. Despite the knowledge available regarding specific residues required for ATP binding and hydrolysis, there has been no report characterizing the specificity of different nucleotide usage exhibited by LT. In this report, we performed a systematic biochemical analysis to investigate the roles of the various amino acid residues around the NTP binding pocket responsible for conferring nucleotide specificity in LT dsDNA unwinding.

## Methods

### Cloning, mutagenesis, expression and purification of LT

The LT 131–627 sequence was cloned into a pGEX-6p-1 vector with a GST fusion at the N-terminus of the truncated protein. The resulting construct was used for site-directed mutagenesis to generate all the mutants used in this study. All mutations were confirmed with sequencing. LT WT and mutant proteins were expressed in *Escherichia coli* and purified via glutathione affinity, precision protease cleavage, further ion exchange and Superdex 200 size exclusion chromatography and quantified as described previously [26]. All LT proteins were then concentrated to 5–10 mg/mL in buffer containing 25 mM Tris-Cl pH 8.0, 250 mM NaCl, and 0.5 mM TCEP and stored at −80 °C as 20 μl aliquots.

### Helicase and oligomerization assays

Helicase assays were performed as described in [26, 27]. The partial dsDNA substrate was obtained by annealing a 3’-6FAM labeled 25-nt random sequence oligonucleotide to a 45-nt oligonucleotide, where only 25 nt of the duplex are complimentary, leaving the unlabeled strand with a 20 nt poly-T 3’extension. Approximately 100 fmol of substrate was incubated with 90 nM of total LT protein in helicase buffer containing 20 mM Tris-Cl pH 7.5, 10 mM MgCl_2_, 0.5 mM NTP, 1 mM DTT, and 0.1 mg/mL BSA for 45 min at 37 °C. The reaction was terminated by adding 0.5% SDS, 25 mM EDTA and 10% glycerol and analyzed using an 8% native polyacrylamide gel in 1X TBE buffer. Gels were scanned by fluorescence and data analysis was done using Quantity One and ImageQuant TL. The oligomerization assays were performed as described in [23] using equal amounts of WT and mutant proteins (0.8 μg/μL, 250 μg total protein in 300uL volume) in each NTP condition with or without Mg^++^. The areas under each peak were then quantified using peak integration in UNICORN.

### Isothermal titration calorimetry assay

ITC experiments were performed at 25 °C using a MicroCal PEAQ-ITC manual-load system (Malvern Instruments). Protein samples of WT LT and LT mutants suspended in storage buffer (25 mM Tris-Cl pH 8.0, 250 mM NaCl and 0.5 mM TCEP) were filled in the sample cell (25 μM at 280 μL volume) and titrated with either 400 μM or 800 μM concentrations of NTP dissolved in the same buffer with or without Mg^++^. Raw titrations curves and ΔQ changes were then recorded and processed using the MicroCal PEAQ-ITC Analysis software and a one set of sites model for binding was implemented for fitting.

## Results

### Generation of LT nucleotide binding pocket mutants

**Fig. 2:**
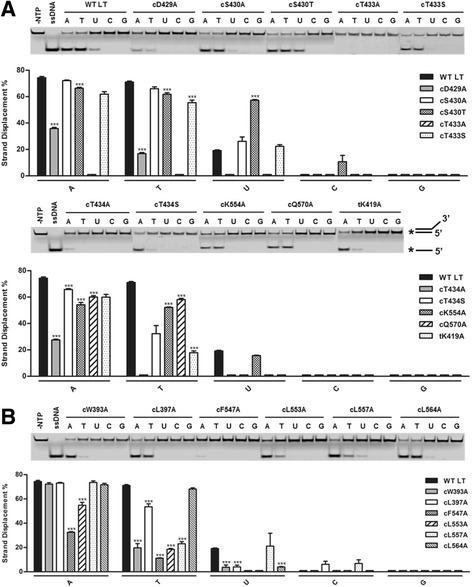
Helicase assay results of LT nucleotide pocket mutants. **a**, **b** The helicase activity assay results for the polar/charged residue mutants **a** and hydrophobic residue mutants **b** are shown in gel analysis data and quantitative bar charts. The native gels shown are a single representation from a set of three individual experiments. Each *lane* (A: ATP, T: TTP, U: UTP, C: CTP, and G: GTP) contains 0.5 mM of each respective NTP, 100 fmol of a 3’-6FAM labeled dsDNA substrate, 90 nM of each respective LT mutant. The ─NTP controls undergo the same reaction without NTP; the ssDNA lane contains the non-annealed labeled ssDNA as the unwound ssDNA control. The *lower* bands indicate the unwound labeled ssDNA strand. The bar graphs represent the average of three individual experiments in the percentage of strand displacement. Changes were calculated via unpaired t-tests and Bonferroni corrected (*n* = 16) with a *p*-value of 0.0031 as threshold for statistical significance, noted with ***

With the high resolution crystal structures of the LT replicative helicase domain in its different nucleotide bound states [15], we identified a group of amino acid residues in the binding pocket within proximity to the sugar and base of the bound nucleotide to perform our systematic analysis of their roles for helicase activity (Fig. 1b). It has been suggested from studies of T7 gp4, a Rec-A type prokaryotic hexameric replicative helicase, that residues positioned closer to the base of the bound nucleotide are more likely to participate in nucleotide specificity [28]. In order to determine which residue(s) in the LT NTP binding pocket play a role in nucleotide specificity for unwinding dsDNA, we designed thirteen alanine and three serine/threonine mutations on a LT 131–627 construct (Fig. 1c–e) to test for their ability to unwind dsDNA substrate using ATP, TTP, UTP, CTP, and GTP. The substrate itself is composed of a labeled 25-mer oligonucleotide annealed to an unlabeled 45-mer oligonucleotide (Fig. 2a), yielding a 25 bp dsDNA segment and a 20 nt poly-thymidine 3’-overhang [24, 29]. The LT 131–627 construct was used since it embodies the functional helicase of LT and has previously been demonstrated to unwind dsDNA with similar efficiency as full-length LT [14, 30].

### Helicase activity of the nucleotide pocket mutants

The helicase assays of the various LT mutants, shown in Fig. 2, summarize the unwinding activities taken from three independent experiments. Out of the group of sixteen LT NTP pocket mutants, four demonstrated altered NTP specificity in unwinding dsDNA. Three of these mutants, tK419A, cW393A, and cL557A, retained their ability to unwind dsDNA using ATP at similar levels compared to wild-type (WT), but exhibited a 3-4 fold decrease in their unwinding activity with TTP (Fig. 2a, b). The fourth mutant, cS430T, maintained comparable dsDNA unwinding activity to WT using both ATP and TTP, but revealed a notable 3-fold increase in unwinding with UTP (Fig. 2a). The other LT mutants displayed either a complete absence of dsDNA unwinding activity, limited changes in unwinding activity compared to WT, or no activity change.

**Fig. 3:**
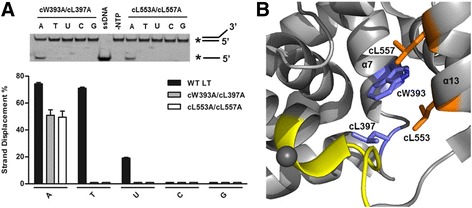
Helicase assay results of the combined double-mutants of hydrophobic residues around the nucleotide pocket. **a** The assay results of the unwinding activities of LT mutants’ cW393A/cL397A and cL553A/cL557A. Conditions for the helicase assays are the same as described in Fig. 2. **b** The position of each set of residues in the nucleotide binding pocket with cW393A/cL397A located on α-helix 7 (*purple*) and cL553A/cL557A on α-helix 13 (*orange*) of the LT hexameric helicase structure. P-loop is colored in yellow as a point of reference

Because of the clear involvement of two hydrophobic residues, cW393 and cL557, in TTP specificity for LT dsDNA unwinding in our results, we also decided to generate double-mutants of these two residues with other hydrophobic residues lining around the nucleotide base area to further investigate their role in the nucleotide specificity of LT. As seen in Fig. 3, mutants cW393A/cL397A and cL553A/cL557A still displayed about 80% of WT activity in unwinding dsDNA with ATP; however, both double-mutants completely lost their ability to carry out its helicase activity with TTP or UTP, confirming the importance of these hydrophobic residues in LT nucleotide specificity. Other double-mutant combinations of these hydrophobic residues were created, but did not express or were not soluble, which preclude them for further analysis (data not shown), possibly due to incorrect folding or decreased stability of these mutations.

The above results of the LT helicase activity show that, among the sixteen pocket residues under investigation, tK419, cW393, and cL557 play an important role in the ability of LT to unwind dsDNA with TTP, and that cS430 appears to function in a more regulatory role in using UTP for LT DNA helicase activity. In order to further understand the molecular basis of these residues in regulating nucleotide specificity for helicase activity, we characterized the biochemical/biophysical properties of the mutant constructs of these four amino acid residues—cS430, tK419, cW393, and cL557.

### Oligomerization properties of LT mutants by size exclusion chromatography (SEC) assay

**Fig. 4:**
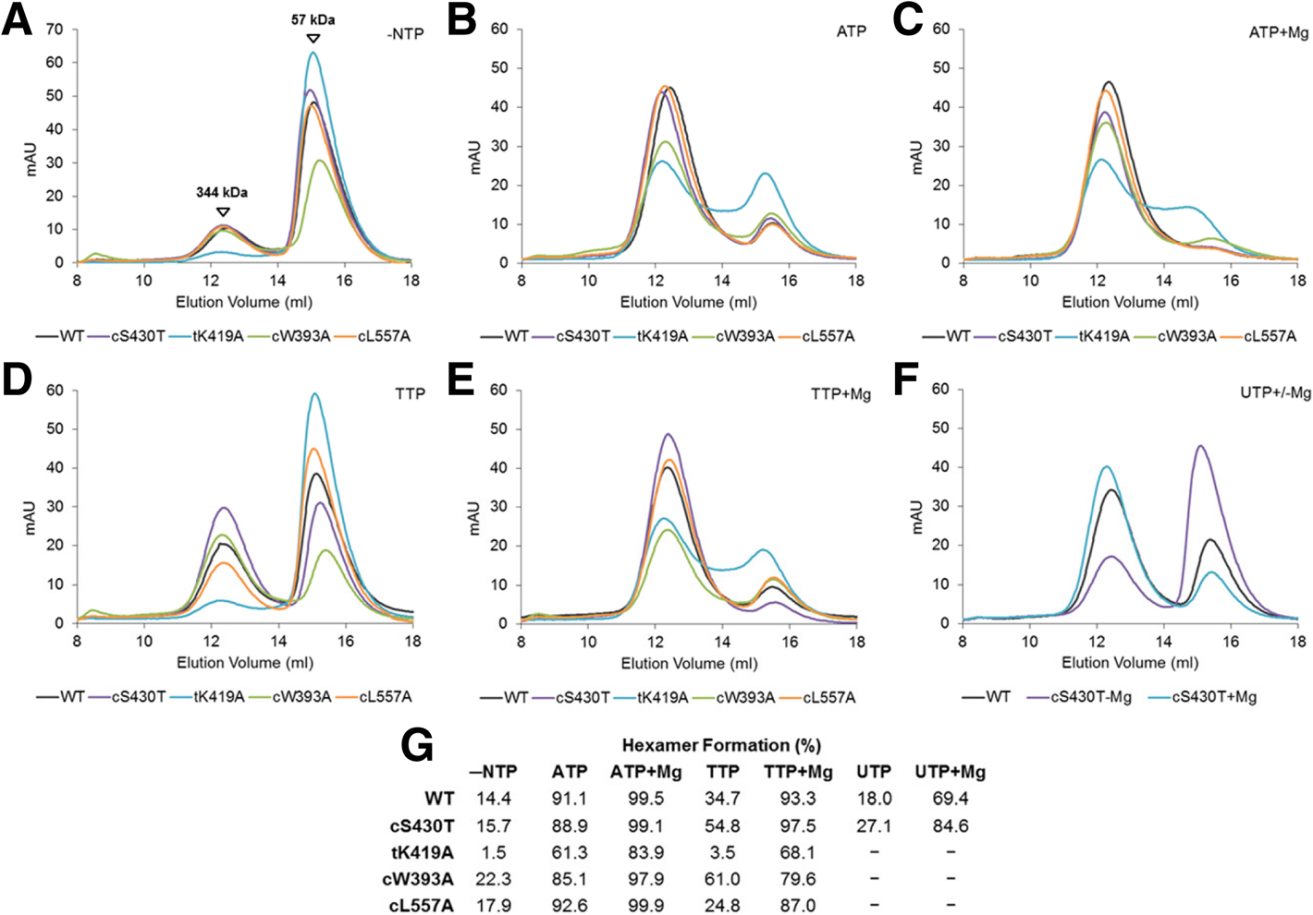
Oligomerization assay of LT WT and nucleotide pocket mutants using gel filtration chromatography on Superdex 200 column. **a**–**f** Gel filtration profiles of WT, cS430T, tK419A, cW393A, and cL557A without NTP (**a**) with ATP (**b**) with ATP + Mg^++^ (**c**) with TTP (**d**) with TTP + Mg^++^ (**e**) and with UTP ± Mg^++^ (**f**). The assays were performed by analyzing 250 μg of LT protein with or without the mentioned NTP and Mg^++^ using Superdex 200 10/300 GL column. The two peaks represent the hexamer (~344 kDa) and monomer (~57 kDa) of LT. **g** The percentages of hexamer formation which were calculated based on the areas under each UV absorption peak in panels A–F. “─” indicates that the oligomerization test in the presence of UTP ± Mg^++^ was not performed. The running buffer contained 25 mM Tris pH 8.0, 250 mM NaCl, and 0.5 mM TCEP. Each protein was incubated with 4 mM NTP and 1 mM MgCl_2_ where indicated

It is known that in order for LT to unwind dsDNA, it needs to oligomerize into ring-shaped hexamers, a process that is greatly enhanced by ATP and Mg^++^ binding. To discern whether the observation that these four residue mutants, tK419A, cW393A, cL557A, and cS430A selectively impact TTP and UTP usage much more than ATP for helicase activity correlates to their hexamerization ability, we performed oligomerization assays of WT LT and each mutant in the presence or absence of ATP, TTP, or UTP with and without Mg^++^ using size exclusion chromatography (SEC). Because CTP/GTP did not support substantial DNA helicase activity for WT and mutant LT (Fig. 2), CTP/GTP were excluded for this and later described studies. For the SEC assay, each LT mutant (approximately 0.8 μg/μL, 250 μg total protein) was incubated with 4 mM of each nucleotide and injected onto a Superdex 200 analytical column in the presence or absence of 1 mM MgCl_2_. The resulting SEC chromatograms are shown in Fig. 4a–f. The areas under each peak were calculated and the percentages of hexamer formed versus monomer by each mutant under each condition are shown in Fig. 4g.

The three LT mutants, tK419A, cW393A, and cL557A that did not affect ATP usage for DNA helicase activity but selectively decreased the efficiency of TTP usage for helicase activity, all showed decreases in hexamer formation in the presence of TTP + Mg^++^ compared to WT, with tK419A showing the largest decrease of 25.2% (Fig. 4g). However, in the presence of ATP + Mg^++^, single mutants’ cW393A and cL557A behaved similarly to WT, maintaining high percentages (97.9 and 99.9%, respectively) of hexamer formation with the exception of tK419A at 83.9% (Fig. 4g). For cS430T that did not affect ATP/TTP usage but selectively increased UTP efficiency for helicase activity, the hexamerization results showed this mutant had almost the same efficiencies in forming hexamers as the WT in the presence of ATP + Mg^++^ and TTP + Mg^++^, but displayed about 15% more hexamer formation than the WT (84.6% for cS430T versus 69.4% for WT) with UTP + Mg^++^ (Fig. 4g). These hexamerization assay results showed a general correlation between the helicase activities of these four mutants and their hexamer formation in the presence of the particular nucleotide demonstrating selectivity by the respective mutant.

### Nucleotide binding properties of LT mutants assayed by ITC

**Fig. 5:**
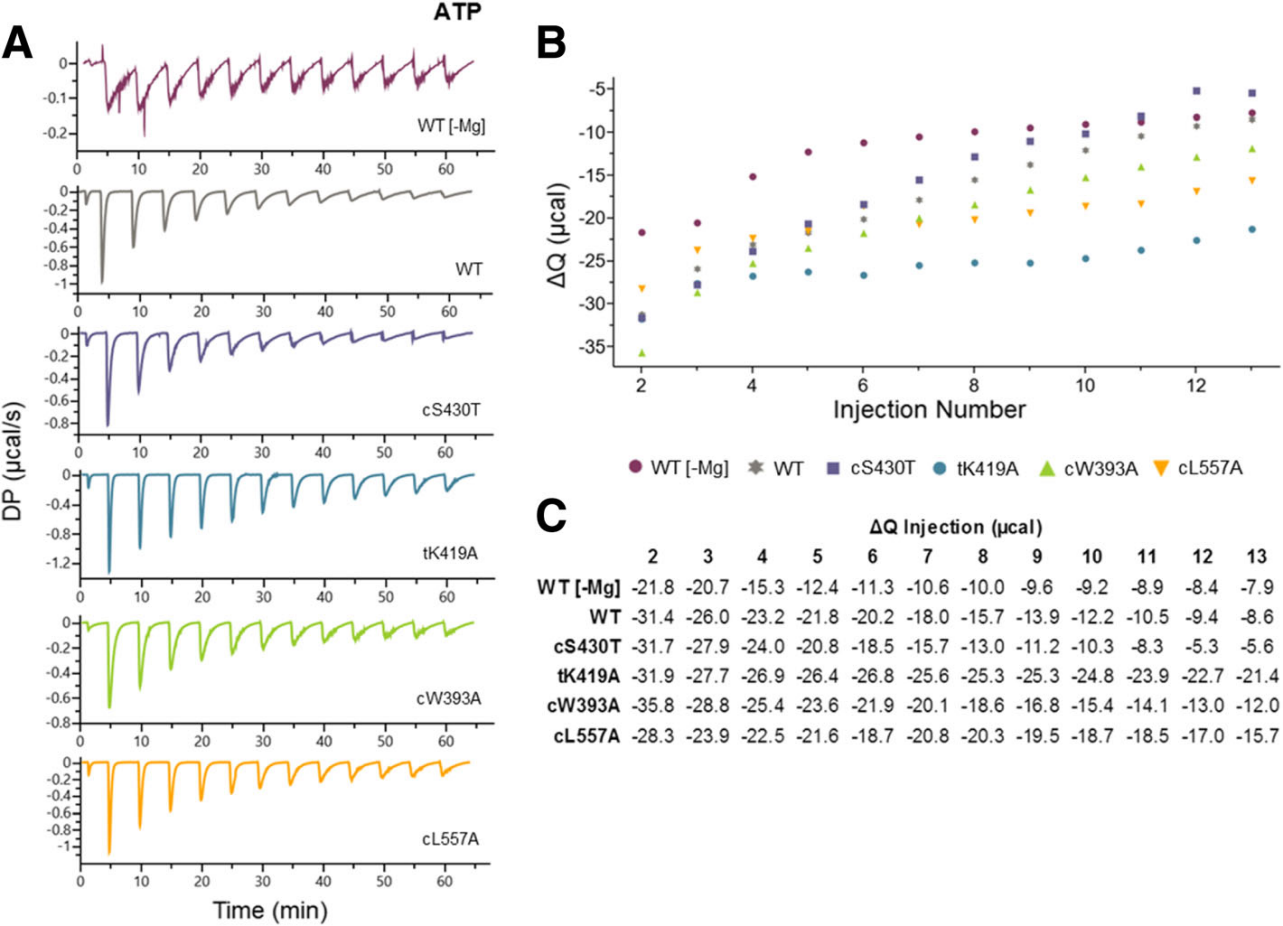
Characterization of ATP binding by LT using isothermal titration calorimetry (ITC). **a** Raw titration curves of WT ± Mg^++^ and different nucleotide pocket mutants in the presence of Mg^++^. Thirteen titrations were measured (μcal/second) for each curve over seventy minutes. **b** Graphical representation of the trends in total heat ΔQ changes over each injection. **c** Quantification of ΔQ over each injection. The negative values indicate an exothermic release of heat. The syringe (ATP) and cell (LT protein) concentrations in each experiment were 400uM and 25uM, respectively and the storage buffer contained 25 mM Tris pH 8.0, 250 mM NaCl, 0.5 mM TCEP, and 1 mM MgCl_2_ unless otherwise noted

**Fig. 6:**
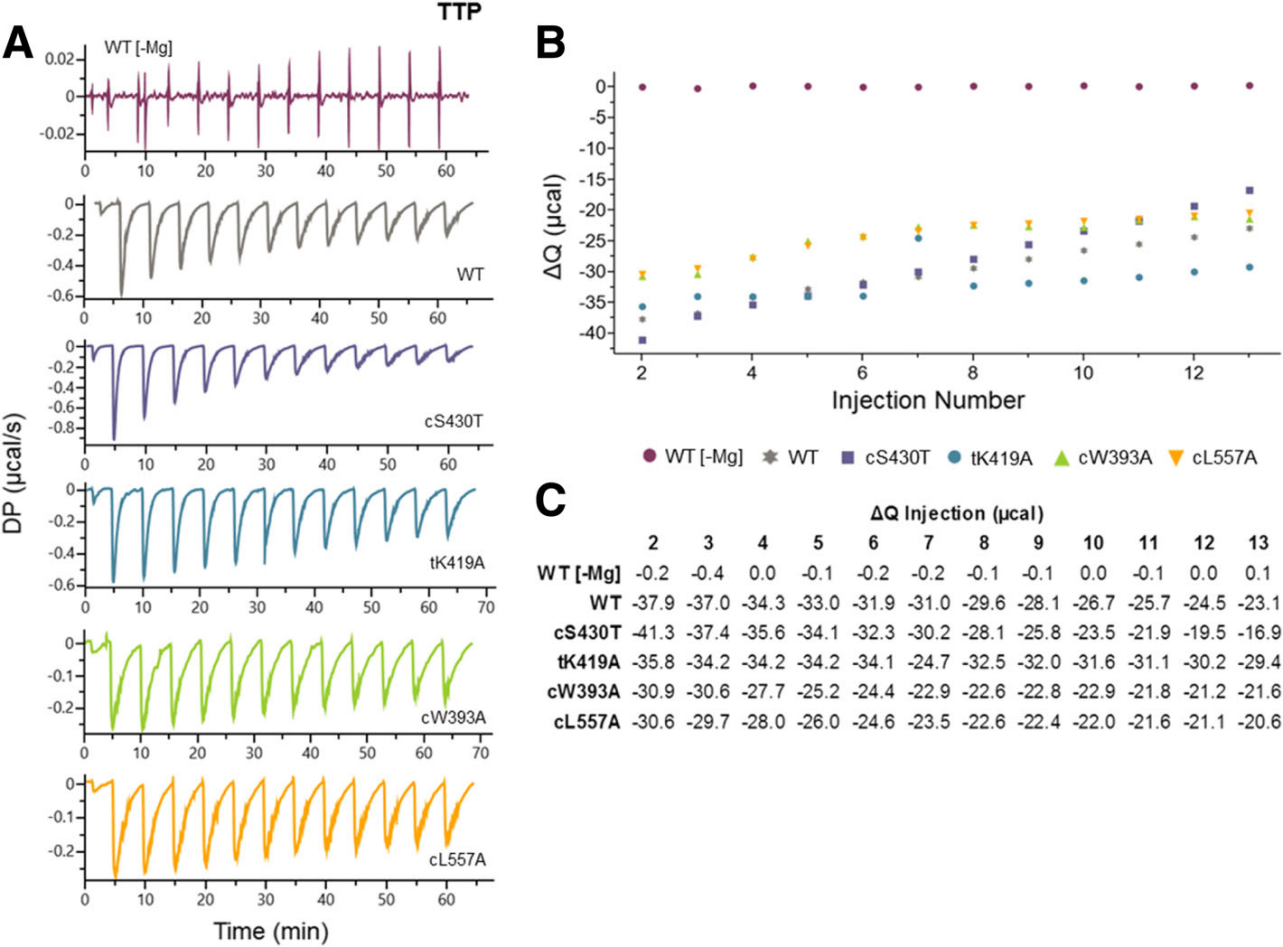
Characterization of TTP binding by LT using ITC. **a** Raw titration curves of WT ± Mg^++^ and different nucleotide pocket mutants in the presence of Mg^++^. Thirteen titrations were measured (μcal/second) for each curve over approximately seventy minutes. **b** Graphical representation of the trends in total heat ΔQ changes over each injection. **c** Quantification of ΔQ over each injection. The negative values indicate an exothermic release of heat. The syringe (TTP) and cell (LT protein) concentrations in each experiment were 800uM and 25uM, respectively and the storage buffer contained 25 mM Tris pH 8.0, 250 mM NaCl, 0.5 mM TCEP, and 1 mM MgCl_2_ unless otherwise noted

**Fig. 7:**
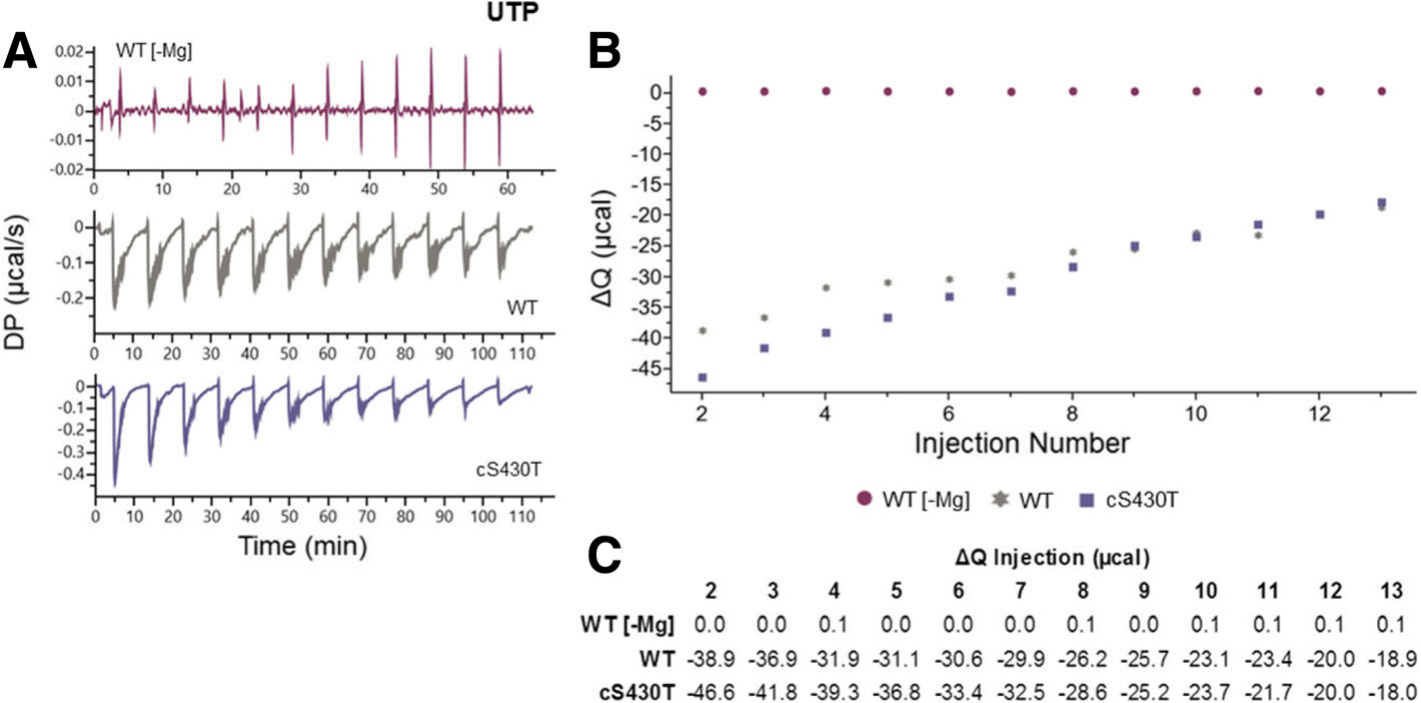
Characterization of UTP binding by LT using ITC. **a** Raw titration curves of WT ± Mg^++^ and different nucleotide pocket mutants in the presence of Mg^++^. Thirteen titrations were measured (μcal/second) for each curve over approximately 70 or 120 min. **b** Graphical representation of the trends in total heat ΔQ changes over each injection. **c** Quantification of ΔQ over each injection. The negative values indicate an exothermic release of heat. The syringe (UTP) and cell (LT protein) concentrations in each experiment were 800uM and 25uM, respectively and the storage buffer contained 25 mM Tris pH 8.0, 250 mM NaCl, 0.5 mM TCEP, and 1 mM MgCl_2_ unless otherwise noted

Previously published work used fluorescently labeled nucleotides to study the interactions of ATP analogs with LT [31]. However, there has been no comprehensive study characterizing the binding of LT to normal nucleotides, such as native ATP or other NTPs, largely due to the challenge of NTP binding being accompanied by the hexamerization and conformational changes of LT. To further understand how these residues confer specificity for each nucleotide during LT dsDNA unwinding, we attempted isothermal titration calorimetry (ITC) to perform a biophysical analysis of the binding properties between each of the four LT mutants and the different nucleotides, with WT LT as a control. The fundamental principle behind ITC is that it measures heat changes that occur when two molecules interact. All heat generated or absorbed, as a result of the two molecules transitioning from an unbound to bound state, are detected using ITC. Because nucleotide binding to LT also triggers oligomerization interactions and conformational changes, each heat response recorded not only signifies NTP binding, but also the inherent hexamer formation and associated conformational changes upon binding, as well as hydrolysis in the presence of Mg^++^. As a result, Kd values for NTP binding to LT could not be deconvoluted from the complex heat responses within the ITC data, reflecting the sum of heat from NTP binding and hydrolysis, hexamerization and conformation changes of LT. However, we found that a careful comparison of the raw ITC data of the NTPs binding by different LT constructs revealed some new and valuable information of LT-NTP binding properties that could not be obtained through other means. The most telling information is represented by the raw ITC titration curves recorded in differential power (DP in μcal/second) and through changes in total heat (ΔQ) generated over each titration in each titration curve (Figs. 5, 6, and 7).

Examining the ATP titration curves for each of the LT mutants with Mg^++^ (Fig. 5a), we observed minimal differences in the initial exothermic release of heat (DP in μcal/second: indicative of strength of binding) for cS430T and cL557A compared to WT, whereas the exothermic releases at each titration were noticeably stronger for tK419A and weaker for cW393A. The trace of each mutant titration curve also resembled that of WT, indicating that each mutant behaved similarly to WT in binding ATP, along with the associated hexamerization, conformational changes, and ATP hydrolysis in the presence of Mg^++^. However, there were noticeable differences in the binding characteristics for three of the four mutants as saturation was reached, giving rise to the variations in ΔQ (Fig.5b, c). Among these three mutants, tK419A exhibited the strongest exothermic releases of heat at almost every titration, particularly from the 4^th^ to 13^th^ titration (Fig. 5c), ending at −21.4 μcal, much higher than ΔQ value of WT at −8.6 μcal. The same trend, to a lesser extent was displayed by cL557A, also resulting in relatively larger ΔQ values at later titrations (Fig. 5c). For mutant cW393A, despite weaker initial exothermic releases of heat at each titration, cW393A exhibited extended periods of equilibration back to the baseline, with the trace of each equilibration period displaying smaller additional releases of heat; its summation over each titration explains the slightly larger ΔQ values compared to WT. These additional releases of heat within the period of equilibration to baseline (after the initial release) likely represent the result of less efficient hexamerization and coordination of hydrolysis, causing additional conformational changes of the complex to occur during both events of the interaction. This same characteristic is observed when ATP is titrated into WT LT without Mg^++^ (Fig. 5a, c). Binding and hexamerization occur due to nucleotide binding, but with no hydrolysis. As a result, the interaction probably produces slow and continuous conformational changes within the complex during each equilibration period of each titration without Mg^++^. Accordingly, even with a 6–7 fold weaker initial exothermic release of heat for ATP without Mg^++^ in the first couple titrations (Fig. 5a), the difference in ΔQ values with and without Mg^++^ in binding ATP are less dramatic over the course of detection (Fig. 5b, c).

When evaluating the titration curves of TTP with each LT construct in the presence of Mg^++^ (Fig. 6a), cW393A and cL557A showed the largest decreases in both the initial exothermic release of heat and the resulting ΔQ values throughout each titration compared to WT (Fig. 6b, c). The trace of each titration curve also exhibited extended periods of equilibration to baseline with additional releases of heat, which may correlate to a higher frequency of additional conformational changes that occur after weakened TTP binding by the mutants. These data suggests that both cW393A and cL557A mutants not only bind TTP with lower affinity, but may also hexamerizes and hydrolyzes TTP with less efficiency compared to WT. On the other hand, cS430T appears to bind TTP with slightly stronger affinity for TTP than WT (Fig. 6a–c), and may also hexamerize and hydrolyze TTP more efficiently compared to WT. This is shown in the trace of its titration curve exhibiting a quicker return to baseline (Fig. 6a). Interestingly, tK419A did not demonstrate significant differences in TTP binding compared to WT (Fig. 6a–c).

For UTP binding by cS430T, which showed much higher unwinding activity than WT using UTP, the initial exothermic release of heat was approximately twice that of WT (Fig. 7a), indicating cS430T binds stronger to UTP compared to WT in the presence of Mg^++^. The action of UTP binding and the accompanying hexamerization of cS430T and hydrolysis of UTP were also stronger for cS430T compared to WT as evident in the traces of their two titration curves (Fig. 7a). Titration of UTP into WT produced longer periods of equilibration to baseline compared to cS430T. The ΔQ values of the first eight titrations also support the stronger UTP binding by the cS430T mutant (Fig. 7b, c). Lastly, it is important to note that with UTP binding by WT LT, the length of time it takes for each titration to equilibrate back to baseline is considerably extended compared with ATP and TTP, with less than 5 mins for ATP and TTP, but more than 10 mins with UTP (compare the time scale of Fig. 7a to that of Figs. 5a and 6a). These results are probably indicative of less efficient hexamerization and slower but prolonged conformational changes when binding to UTP, and possibly less efficient hydrolysis of UTP by LT in the presence of Mg^++^.

### Mg^++^ role in LT oligomerization and NTP binding

When examining WT LT oligomerization by SEC (Fig. 4) and binding by ITC with ATP (Fig. 5) in the presence or absence of Mg^++^, it is interesting to note that while there was only a less than ten percent difference in the ability of WT LT to oligomerize with ATP with/without Mg^++^, there was a drastic difference in the ATP binding characteristics of WT LT with/without Mg^++^. Without Mg^++^, WT LT was still able to efficiently form stable hexamers with ATP (Fig. 4b, g). However, when titrating ATP into WT LT in the absence of Mg^++^, even though binding was evident, the recorded results were substantially weaker and less coordinated than with Mg^++^ (Fig. 5a–c). For TTP and UTP binding by the WT LT, the SEC results correlated more closely with that from the ITC results. Significant increase of hexamerization of LT was observed only when TTP/UTP and Mg++ were both present (Fig. 4g), and significant TTP and UTP binding was only observed in the presence of Mg^++^ (Figs. 6a and 7a).

## Discussion

SV40 LT is a replicative helicase that recognizes origin DNA and assembles into a hexameric/double hexameric helicase to initiate DNA replication of the viral genome. LT is unique in that LT alone can open up the origin dsDNA and efficiently unwind fork DNA, without the help of other protein factors through coupling the energy from ATP binding and hydrolysis. In this report, we described the first comprehensive characterization of the various residues around the nucleotide pocket of the LT hexameric helicase in order to understand their roles in DNA helicase activity. By systematically mutating thirteen residues to generate sixteen mutants around the nucleotide pocket, we showed that twelve mutants either significantly reduced or completely abolished DNA helicase activity. Interestingly, among these sixteen mutants, we have identified four mutants (i.e. cS430, tK419, cW393 and cL557) that selectively displayed a more profound impact on certain nucleotide usage for LT DNA helicase activity. Furthermore, we showed that the four residues mutated in these four mutants play a role in the selective NTP binding and the associated oligomerization of LT.

**Fig. 8:**
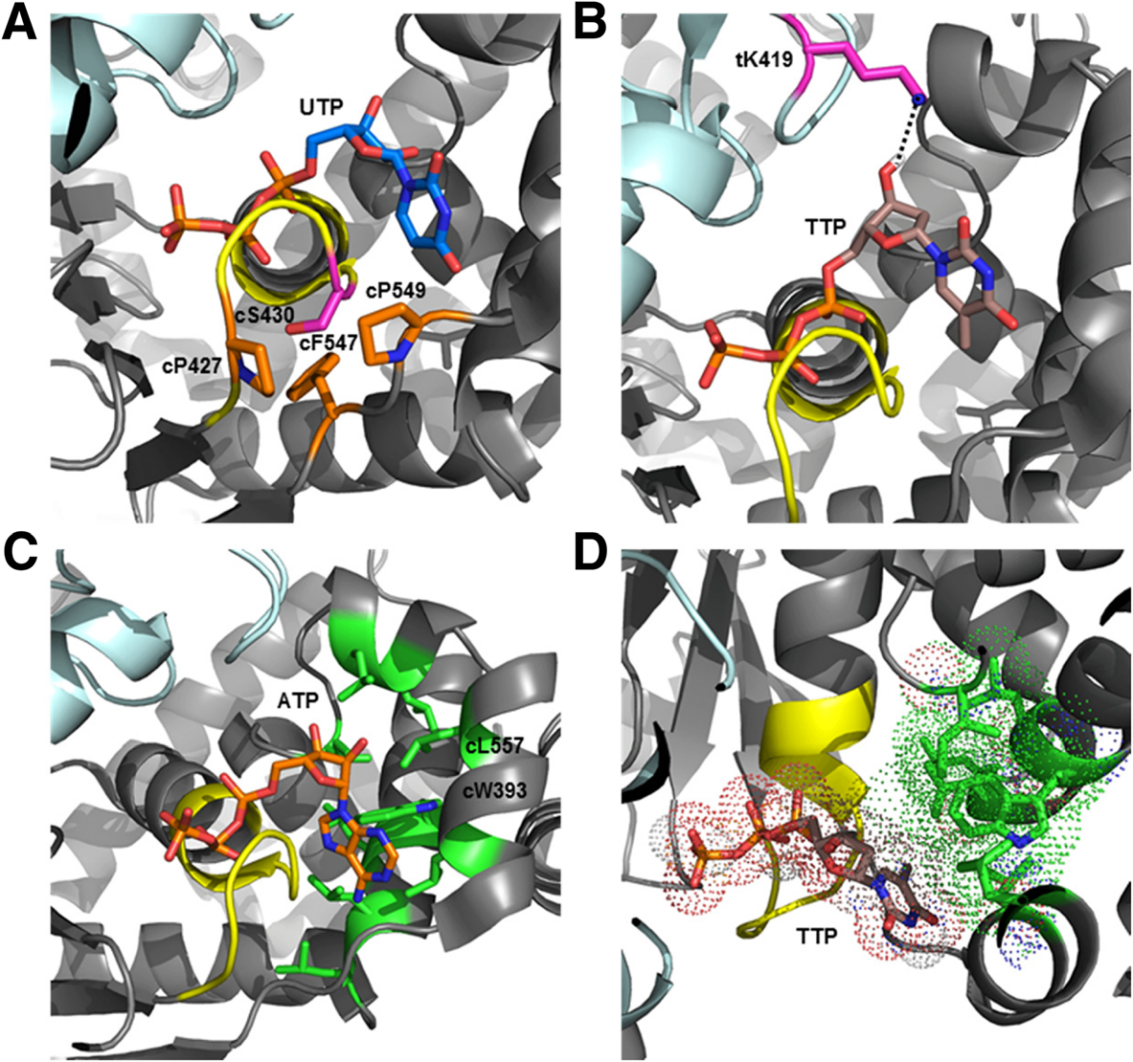
Structural implications of LT nucleotide specificity. **a** Residues cP427, cF547, and cP549 (*orange*) are presented in sticks to demonstrate the rigidity in the spatial arrangement surrounding cS430 (*magenta*). **b** The charge-charge interaction between tK419 (*magenta*) and the -OH group on the sugar molecule of TTP. **c** The unique hydrophobic lining of nine hydrophobic residues (*green*), including cW393 and cL557 around base of the bound nucleotide in the nucleotide pocket of LT. **d** The function of the hydrophobic lining through an induced fit mechanism as illustrated with TTP, in which cW393, cL557 and other hydrophobic residues alter their conformations compared to those in panel-C. TTP and UTP were docked and fitted into the LT nucleotide binding pocket utilizing AutoDock Vina in PyMol. The lowest energy state docking with the correct alignment of the α-β-γ phosphates of the bound NTP was chosen for their respective *panels*

Among the four mutants, cS430T exhibited an unexpected 3-fold increase in unwinding activity with UTP than WT LT, even though its unwinding activities with ATP or TTP were similar to that of WT. Oligomerization by SEC assay showed that cS430T produced 15% more hexamers than WT with UTP + Mg^++^ (Fig. 4g), whereas it yielded similar hexamer percentages as the WT in the presence of ATP + Mg^++^ and TTP + Mg^++^. The residue cS430 is located on the P-loop, right beneath the bound nucleotide and is not expected to interact with the nucleotide directly. Rather, cS430 is surrounded by two rigid proline residues (cP427 and cP549) and an aromatic cF547 (Fig. 8a), and it is possible that the mutation to a larger size threonine with an extra methyl group within that confined space could shift the P-loop upward, providing a tighter fit for binding the single-ring uridine base. Consistent with this hypothesis is that cS430A, a smaller alanine mutation behaved almost identically to WT (Fig. 2a). The hypothesis further aligns with the ITC results that showed stronger interaction of cS430T with UTP than WT (Fig. 7a). The hydroxyl group on the serine residue may also contribute to this specificity as mutating cS430 to a valine residue showed approximately 40, 80, and 60% decreases in unwinding activity with ATP, TTP, and UTP, respectively (data not shown).

Another mutant, tK419A is unique in that it exhibited 54% reduction of helicase activity with TTP and a near complete loss of activity with UTP, even though it retained most (approx. 80%) of WT activity with ATP (Fig. 2a). Interestingly, tK419A showed consistent reductions in the degree of hexamer formation with or without ATP or TTP and Mg^++^ compared to WT. tK419 directly interacts with the -OH group of the sugar moiety of the bound ATP [15], and likely other nucleotides (Fig. 8b). Therefore, the selective reduction of helicase activity with TTP and UTP may not be directly correlated with the charge-charge interaction of tK419 with the sugar -OH. However, it is possible that interactions made by the bound ATP, TTP, or UTP at the base moiety could be slightly different from each other due to subtle differences in structure, size, and positioning within the binding pocket, making the interaction between tK419 and TTP/UTP more important in their binding and hydrolysis than with ATP. This may explain why the helicase activity and oligomerization of tK419A with TTP/UTP are selectively much more affected than with ATP.

Nucleotide specificity has also been studied previously with the bacteriophage T7 gp4 hexameric helicase that uses dTTP as its primary energy source for unwinding DNA [28]. The study identified three polar/charged residues around the nucleotide pocket of T7 gp4 that play a role in nucleotide specificity, including a gain of unwinding activity using dCTP/dGTP for a S319T mutation, a shift of preference from dTTP to dATP usage for unwinding through an R504A mutation, and selectively decreasing dATP usage without drastically affecting dTTP usage for an R363A mutation [28]. In contrast, we did not observe any gain of unwinding activity using CTP/GTP or any dramatic switch from TTP to ATP or vice versa from any of the LT mutations in our study, even though the equivalent residues of S319 and R504 in T7 gp4 were also investigated in LT here (i.e. cT433 and cK454, Figs. 1d and 2a). One caveat is that we tested ATP/CTP/GTP instead of dATP/dCTP/dGTP for the LT constructs used in this study as there may be potential differences in unwinding efficiency between LT utilization of nucleotide versus deoxynucleotide. However, it has been demonstrated that WT LT uses ATP, CTP, and GTP with almost identical efficiency in unwinding duplex DNA as with each deoxyribose equivalent [24].

Additionally, the LT hexameric helicase has a unique structural feature at the nucleotide pocket where several hydrophobic residues together form a hydrophobic lining around the base of the bound nucleotide (Figs. 1e and 8c). This highly hydrophobic enveloped nucleotide pocket is not present in any of the other structurally characterized ring-shaped hexameric helicases/translocases such as DnaB [32], E1 [33], T7 gp4 [34], archaeal MCM [35], or RuvB [36] and LT residues cW393 and cL557 are at the core of this hydrophobic lining. Single mutants of these two residues, cW393A and cL557A, did not affect their ability to unwind dsDNA with ATP, but displayed a 3-4 fold decrease in unwinding with TTP (Fig. 2b). Consistent with this change of helicase activity is that cW393A and cL557A were able to efficiently form stable hexamers in the presence of ATP, but showed reductions in the degree of hexamer formation with TTP in the presence/absence of Mg^++^ (Fig. 4g). Moreover, both mutants displayed weaker binding and associated coordination and hydrolysis of TTP through ITC studies (Fig. 6a).

The multiple hydrophobic residues at the nucleotide pocket of LT work together to pack with the base moiety of ATP (Fig. 8c) and likely with other nucleotides as well, such as TTP. When packing with ATP and its larger purine base, its interactions with these multiple hydrophobic residues may be sufficiently strong such that cW393 can be mutated to alanine without having an obvious effect on ATP binding or usage for helicase activity. However, when packing with TTP (or UTP), the interactions between the smaller pyrimidine base of TTP/UTP and the hydrophobic residues likely relies more heavily on the larger aromatic cW393 residue for TTP/UTP to be stably bound for efficient binding and hydrolysis. This may explain why the cW393A mutation selectively impacts TTP usage in DNA unwinding activity. A similar rationale goes for the cL557A mutant. In addition to its own interactions with TTP, cL557 also packs with cW393, and is expected to be important for stabilizing the conformation of cW393 for packing with the smaller pyrimidine ring of TTP. Therefore, cL557A is also expected to selectively affect TTP binding and usage much more than affecting ATP.

In addition to cW393 and cL557, the unique architecture of multiple hydrophobic lining in the NTP binding pocket of LT consists of six other leucines (cL394, cL397, cL398, cL553, cL563, cL564) and one isoleucine (cI569) residue surrounding the nucleotide base (Fig. 8c, d). Therefore, to determine whether this structural feature plays a role in nucleotide specificity, we performed a double-mutant analysis of the four residues (cW393, cL397, cL553, cL557) near the purine base of ATP as shown in the co-crystal structure (PDBid: 1SVM), generating double-mutants cW393A/cL397A and cL553A/cL557A to attempt to further understand the broader effects of this hydrophobic lining. Helicase assays revealed that each double-mutant displayed about a 25% decrease of activity using ATP, but showed no detectible activity using TTP and UTP. The results further confirm the role of these multiple hydrophobic residues and their packing interactions with the larger purine base of ATP, which can afford the loss of one hydrophobic residue without majorly impacting binding. However, in their packing interactions with the smaller pyrimidine base of TTP/UTP, which is weaker, the loss of a single hydrophobic residue (such as cW393 or cL557) or two such residues (such as the double-mutants) can significantly disturb the binding and usage of TTP/UTP in LT DNA helicase activity.

The role of Mg^++^ has been well studied in other replicative hexameric helicases such as DnaB and T7 gp4. In DnaB, the presence of Mg^++^ stabilizes the formation of hexamers for unwinding activity, where in its absence, it exists only as trimers that dissociate into monomers [37]. In contrast, for T7 gp4, Mg^++^ is not a prerequisite for either hexamer formation or dTTP binding [38]. When considering the oligomerization results of each LT protein (WT and mutants) in this study, with or without Mg^++^, the divalent ion appears to function in a more important role with TTP and UTP compared to with ATP. Every LT protein exhibited substantial reductions in the degree of hexamer formation in the absence of Mg^++^ with TTP and UTP, while the majority of proteins showed almost identical degrees of hexamer formation in the presence/absence of Mg^++^ with ATP. Interestingly, as seen in the ITC study of the binding characteristics between WT LT and each of the three nucleotides, while the overall trend for ATP binding was much less dependent on Mg^++^, ITC results revealed absolutely no binding for TTP and UTP without the presence of Mg^++^. To our knowledge, this data is the first to show a selective role of Mg^++^ affecting the nucleotide binding and oligomerization properties of a replicative hexameric helicase depending on the specific nucleotide bound. For LT, Mg^++^ plays a more critical role in the stabilization of TTP and UTP within each hexameric interface for efficient binding and oligomerization than compared to with ATP.

## Conclusion

In summary, our systematic mutational analysis of the LT nucleotide binding pocket identified four key residues (tK419, cW393, cL557, and cS430) within the binding pocket determining TTP and UTP specificity. The study provides a first look into the specific interactions contributing to the selective usage of nucleotides by LT in its DNA helicase activity.

## Abbreviations

AAA+: *A*TPase *a*ssociated with various cellular *a*ctivities
ATP: Adenosine triphosphate
CTP: Cytidine triphosphate
GTP: Guanosine triphosphate
ITC: Isothermal titration calorimetry
LT: Large tumor antigen
Mg^++^: Magnesium
NTP: Nucleotide triphosphate
PDBid: Protein Data Bank Identification
SEC: Size exclusion chromatography
SV40: Simian virus 40
T7 gp4: Bacteriophage T7 gene product 4
TTP: Thymidine triphosphate
UTP: Uridine triphosphate
WT: Wild-type
ΔQ: Change in total heat

## References

[1] Singleton MR, Dillingham MS, Wigley DB (2007). Structure and mechanism of helicases and nucleic acid translocases. Annu Rev Biochem.

[2] Fanning E, Knippers R (1992). Structure and function of simian virus 40 large tumor antigen. Annu Rev Biochem.

[3] Ahuja D, Saenz-Robles MT, Pipas JM (2005). SV40 large T antigen targets multiple cellular pathways to elicit cellular transformation. Oncogene.

[4] Sullivan CS, Pipas JM (2002). T antigens of simian virus 40: molecular chaperones for viral replication and tumorigenesis. Microbiol Mol Biol Rev.

[5] Cheng J, Decaprio JA, Fluck MM, Schaffhausen BS (2009). Cellular transformation by Simian Virus 40 and Murine Polyoma Virus T antigens. Semin Cancer Biol.

[6] Simmons DT (2000). SV40 large T antigen functions in DNA replication and transformation. Adv Virus Res.

[7] Pipas JM (2009). SV40: cell transformation and tumorigenesis. Virology.

[8] Borowiec JA, Dean FB, Bullock PA, Hurwitz J (1990). Binding and unwinding—how T antigen engages the SV40 origin of DNA replication. Cell.

[9] Stahl H, Droge P, Knippers R (1986). DNA helicase activity of SV40 large tumor antigen. EMBO J.

[10] Gai D, Chang YP, Chen XS (2010). Origin DNA melting and unwinding in DNA replication. Curr Opin Struct Biol.

[11] Scheffner M, Knippers R, Stahl H (1989). RNA unwinding activity of SV40 large T antigen. Cell.

[12] Bullock PA (1997). The initiation of simian virus 40 DNA replication in vitro. Crit Rev Biochem Mol Biol.

[13] Wun-Kim K, Simmons DT (1990). Mapping of helicase and helicase substrate-binding domains on simian virus 40 large T antigen. J Virol.

[14] Gai D, Li D, Finkielstein CV, Ott RD, Taneja P, Fanning E (2004). Insights into the oligomeric states, conformational changes, and helicase activities of SV40 large tumor antigen. J Biol Chem.

[15] Gai D, Zhao R, Li D, Finkielstein CV, Chen XS (2004). Mechanisms of conformational change for a replicative hexameric helicase of SV40 large tumor antigen. Cell.

[16] Arthur AK, Hoss A, Fanning E (1988). Expression of simian virus 40 T antigen in Escherichia coli: localization of T-antigen origin DNA-binding domain to within 129 amino acids. J Virol.

[17] Li D, Zhao R, Lilyestrom W, Gai D, Zhang R, Decaprio JA (2003). Structure of the replicative helicase of the oncoprotein SV40 large tumour antigen. Nature.

[18] Koonin EV (1993). A common set of conserved motifs in a vast variety of putative nucleic acid-dependent ATPases including MCM proteins involved in the initiation of eukaryotic DNA replication. Nucleic Acids Res.

[19] Tjian R, Robbins A (1979). Enzymatic activities associated with a purified simian virus 40 T antigen-related protein. Proc Natl Acad Sci U S A.

[20] Fanning E (1992). Simian virus 40 large T antigen: the puzzle, the pieces, and the emerging picture. J Virol.

[21] Dean FB, Bullock P, Murakami Y, Wobbe CR, Weissbach L, Hurwitz J (1987). Simian virus 40 (SV40) DNA replication: SV40 large T antigen unwinds DNA containing the SV40 origin of replication. Proc Natl Acad Sci U S A.

[22] Mastrangelo IA, Hough PV, Wall JS, Dodson M, Dean FB, Hurwitz J (1989). ATP-dependent assembly of double hexamers of SV40 T antigen at the viral origin of DNA replication. Nature.

[23] Greenleaf WB, Shen J, Gai D, Chen XS (2008). Systematic study of the functions for the residues around the nucleotide pocket in simian virus 40 AAA+ hexameric helicase. J Virol.

[24] Wiekowski M, Schwarz MW, Stahl H (1988). Simian virus 40 large T antigen DNA helicase. Characterization of the ATPase-dependent DNA unwinding activity and its substrate requirements. J Biol Chem.

[25] Lorimer HE, Wang EH, Prives C (1991). The DNA-binding properties of polyomavirus large T antigen are altered by ATP and other nucleotides. J Virol.

[26] Shen J, Gai D, Patrick A, Greenleaf WB, Chen XS (2005). The roles of the residues on the channel beta-hairpin and loop structures of simian virus 40 hexameric helicase. Proc Natl Acad Sci U S A.

[27] Yu XJ, Greenleaf WB, Shi YS, Chen XS (2015). Mechanism of subunit coordination of an AAA+ hexameric molecular nanomachine. Nanomedicine.

[28] Satapathy AK, Crampton DJ, Beauchamp BB, Richardson CC (2009). Promiscuous usage of nucleotides by the DNA helicase of bacteriophage T7: determinants of nucleotide specificity. J Biol Chem.

[29] Goetz GS, Dean FB, Hurwitz J, Matson SW (1988). The unwinding of duplex regions in DNA by the simian virus 40 large tumor antigen-associated DNA helicase activity. J Biol Chem.

[30] Gai D, Wang D, Li SX, Chen XS (2016). The structure of SV40 large T hexameric helicase in complex with AT-rich origin DNA. Elife.

[31] Huang SG, Weisshart K, Fanning E (1998). Characterization of the nucleotide binding properties of SV40 T antigen using fluorescent 3'(2')-O-(2,4,6-trinitrophenyl)adenine nucleotide analogues. Biochemistry.

[32] Bailey S, Eliason WK, Steitz TA (2007). Structure of hexameric DnaB helicase and its complex with a domain of DnaG primase. Science.

[33] Enemark EJ, Joshua-Tor L (2006). Mechanism of DNA translocation in a replicative hexameric helicase. Nature.

[34] Sawaya MR, Guo S, Tabor S, Richardson CC, Ellenberger T (1999). Crystal structure of the helicase domain from the replicative helicase-primase of bacteriophage T7. Cell.

[35] Brewster AS, Wang G, Yu X, Greenleaf WB, Carazo JM, Tjajadi M (2008). Crystal structure of a near-full-length archaeal MCM: functional insights for an AAA+ hexameric helicase. Proc Natl Acad Sci U S A.

[36] Putnam CD, Clancy SB, Tsuruta H, Gonzalez S, Wetmur JG, Tainer JA (2001). Structure and mechanism of the RuvB Holliday junction branch migration motor. J Mol Biol.

[37] Bujalowski W, Klonowska MM, Jezewska MJ (1994). Oligomeric structure of Escherichia coli primary replicative helicase DnaB protein. J Biol Chem.

[38] Picha KM, Patel SS (1998). Bacteriophage T7 DNA helicase binds dTTP, forms hexamers, and binds DNA in the absence of Mg2+. The presence of dTTP is sufficient for hexamer formation and DNA binding. J Biol Chem.

